# Pseudopancreatitis on computed tomography in a patient with isolated blunt head trauma: a case report

**DOI:** 10.1186/1752-1947-8-56

**Published:** 2014-02-16

**Authors:** Ah-Ling Cheng, Eddy S Lang

**Affiliations:** 1 Department of Diagnostic Imaging, University of Calgary, Foothills Medical Centre, 1403 – 29 Street, NW, Calgary, AB T2N 2T9, Canada; 2 Department of Emergency Medicine, University of Calgary, Foothills Medical Centre, 1403 – 29 Street, NW, Calgary, AB T2N 2T9, Canada

**Keywords:** Neurogenic shock, Pancreatic trauma, Pseudopancreatitis, Shock pancreas

## Abstract

**Introduction:**

Computed tomography is commonly used to exclude occult injuries in patients with trauma, but imaging can reveal findings that are of uncertain etiology or clinical significance. We present a case of unsuspected pancreatic abnormality in a female patient with trauma who sustained an isolated blunt head injury.

**Case presentation:**

A 25-year-old female Caucasian patient sustained massive blunt and penetrating head trauma, secondary to a large object penetrating through the vehicle windshield. Based on the mechanism of injury and clinical evaluation, it was felt to be an isolated head injury. However, computed tomography of her abdomen revealed an occult, intra-abdominal finding of significant pancreatic enlargement and peripancreatic fluid. There was no computed tomography evidence of parenchymal pancreatic laceration. The appearance of her pancreas on computed tomography was identical to that of acute pancreatitis or low-grade pancreatic injury, but her clinical history and laboratory values were not consistent with this, hence the term ‘pseudopancreatitis’. Later surgery for organ donation confirmed diffuse pancreatic and peripancreatic edema, but no hematoma, contusion or other evidence for direct traumatic injury. This was an isolated intra-abdominal abnormality.

**Conclusion:**

The routine use of computed tomography in patients who have sustained trauma has led to increasing detection of unexpected findings. Clinical information such as mechanism of injury and blood work, along with careful evaluation of ancillary imaging findings (or lack of), is important for the provision of an appropriate differential diagnosis. We discuss the possible mechanism and differential diagnosis of an isolated pancreatic abnormality in the setting of non-abdominal trauma, which includes shock pancreas, overhydration, traumatic pancreatic injury and pancreatitis secondary to other etiologies.

## Introduction

Traumatic injury of the pancreas is unusual, and isolated pancreatic injury is rare [1-3]. Trauma to the pancreas can result in secondary pancreatitis, which can be a clinical, biochemical and imaging diagnosis. However, if there is no evidence of direct abdominal trauma, determining the etiology and clinical significance of an abnormal pancreatic appearance on imaging may be difficult in the acute care setting.

## Case presentation

A 25-year-old Caucasian woman was brought to our emergency department after a single, high velocity projectile penetrated her vehicle windshield and struck her in the head. The vehicle was found pulled to the side of the road with transmission in park; there was no evidence of vehicular damage apart from the windshield damage. It was presumed by emergency responders that the patient had pulled over and stopped the car. The patient had been wearing a seatbelt. On presentation to our emergency department, our patient had a Glasgow Coma Scale score of 3, and was subsequently intubated for airway protection. A physical examination revealed a blood pressure of 111/87mmHg and her heart rate ranged from 100 to 150 beats per minute. A neurological examination revealed fixed and dilated pupils, as well as decerebrate posturing. She had a large, bleeding scalp laceration but no foreign material was noted. The remainder of the physical examination was normal. No other contusions or lacerations of her neck, chest or abdomen were identified. Of note, there was absence of a lap belt sign.

**Figure 1:**
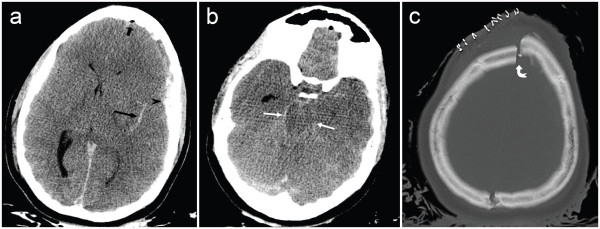
**Computed tomography images of the patient’s head. (a, b)** Unenhanced axial computed tomography images demonstrate subdural (arrowhead) and subarachnoid hemorrhage (long black arrow), pneumocephalus (short black arrow), midline shift to the right, and transtentorial herniation (white arrows). **(c)** Numerous skull fractures were present, including a fracture through the sagittal suture with diastasis through the midline skull (curved white arrow).

Initial laboratory results showed a normal hemoglobin level of 130g/dL and hematocrit of 0.40. A toxicology screen, including alcohol, was negative. A subsequent total body computed tomography (CT) scan, performed approximately 1 hour 15 minutes after the accident, revealed extensive intracranial injuries, including acute subdural hematoma, subarachnoid hemorrhage and cerebral edema, with loss of gray-white matter differentiation (Figure 1). Transtentorial and cerebellar tonsillar herniation were also present. There were numerous skull fractures, including splitting and diastasis of her skull through the sagittal suture and the left lambdoidal suture (Figure 1). A depressed fracture of the frontal bone, with associated pneumocephalus, was also present, in addition to a fracture through her planum sphenoidale.

**Figure 2:**
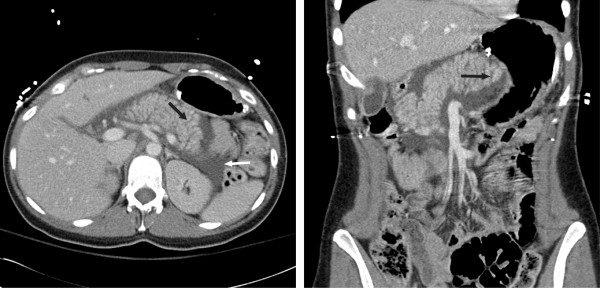
**Enhanced axial and coronal computed tomography images through the abdomen demonstrate diffuse enlargement of the pancreas.** Numerous linear hypodensities in the pancreas have the appearance of widened pancreatic folds (black arrows). Simple fluid is localized to the peripancreatic region, within the retroperitoneum (white arrow).

CT of her abdomen and pelvis with intravenous contrast demonstrated an abnormal pancreas, which appeared diffusely enlarged (Figure 2). Multiple linear hypodensities were seen throughout her pancreas, which had the appearance of prominent pancreatic folds. No pancreatic laceration was identified. A small to moderate amount of simple fluid was present in her abdomen, but localized to her retroperitoneum; specifically, the peripancreatic region. Overall, the findings simulated the CT appearance of early acute pancreatitis or low-grade pancreatic injury, which have overlapping imaging characteristics. However, no other abnormalities, or other findings of traumatic injury, were seen within the remainder of her chest, abdomen or pelvis. In particular, the structures adjacent to her pancreas, including her liver, duodenum, spleen and aorta, were normal. There were no gallstones seen on CT, and there was no intrahepatic biliary duct or common bile duct dilation. The mechanism of our patient’s injury, a single penetrating injury to her head, could not account for the finding.

Our patient was subsequently admitted to our intensive care unit. Her serum lipase levels obtained during this time were within normal limits, at 14U/L (normal range: 0 to 60 U/L), and her amylase levels were elevated at 248U/L (normal range: 30 to 110U/L). A repeat amylase level was not obtained, but a repeat lipase level (obtained approximately six hours later) remained within normal limits at 10U/L. Her neurological status failed to improve and a subsequent perfusion nuclear medicine study showed findings consistent with brain death. Consent was obtained from the patient’s family for organ donation.

During a laparotomy for organ donation, approximately 28 hours from the time of the initial trauma, the transplant surgeon observed marked pancreatic and peripancreatic edema, but no findings consistent with traumatic injury, such as laceration, pancreatic duct injury or regional hemorrhage. This was an isolated finding at surgery, with no other findings of traumatic injury identified in the remainder of her abdomen. Her pancreas was not removed for organ transplant, given its edematous appearance.

## Discussion

We present the case of a patient who presented to our emergency department with isolated and severe head trauma by mechanism, but in whom abdominal imaging revealed an unexpected finding of an abnormal pancreas. The term pseudopancreatitis has been used in a trauma setting where there are CT findings of intra- and peripancreatic fluid (mimicking imaging findings of acute pancreatitis or low-grade pancreatic injury), but in the absence of direct abdominal trauma. To the best of our knowledge, there has only been one other case series, involving four patients, describing similar findings of isolated pancreatic abnormality in patients who have sustained trauma with no abdominal trauma [4]. Those patients, soldiers in the Israel-Lebanon conflict, sustained combat injuries to their extremities, but no abdominal injuries. They were treated with aggressive fluid resuscitation during their prolonged rescue time (mean time 4 hours 45 minutes to first CT). All these patients had decreased hemoglobin values and decreased hematocrit.

One theory postulated by the authors of that case series was overhydration secondary to massive or prolonged fluid resuscitation, where intra- and peripancreatic fluid likely represented extravasated saline and serum within the interstitium of the pancreas. However, by contrast, our patient was not resuscitated for a prolonged period of time before CT was performed, with the time from accident scene to CT scan totaling 1 hour and 15 minutes. Initial laboratory values in our emergency department (obtained just prior to the CT study) showed that our patient’s hemoglobin and hematocrit were within normal limits. The total fluid volume administered to our patient after initial blood work until the CT scan totaled no more than 3L, which included normal saline, packed red blood cells and mannitol. However, many trauma patients receive this volume of fluid during initial resuscitation, without pancreatic changes on CT. Also, whereas other signs of overhydration were noted on the CT studies of the patients in the case series - specifically, fluid tracking along the portal veins in the liver - this was not present in our patient.

In addition to overhydration, another possibility to account for the CT findings was pancreatic trauma. Traumatic pancreatic injury is uncommon, seen in 5% of (blunt) abdominal injury cases, and usually caused by direct trauma to the mid-to-upper abdomen [5]. An isolated pancreatic injury is even more unusual, as up to 70% of patients with pancreatic trauma have associated injuries, most commonly involving the duodenum, stomach, liver and spleen [3]. Depending on the mechanism of injury and the direction of force, the ascending colon, descending colon and kidneys may also be involved [6]. A diagnosis of pancreatic trauma is initially based on a combination of clinical findings, serum lipase or amylase levels, and radiological investigations. CT is currently the initial imaging modality of choice in stable patients, with reported sensitivities up to 85% in the first 24 hours of injury [6]. Endoscopic retrograde cholangiopancreatography (ERCP) and magnetic retrograde cholangiopancreatography (MRCP) can also play a role in diagnosing pancreatic duct injury. MRCP is useful in demonstrating peripancreatic fluid collections and pancreatic ductal anatomy, or disruption of the duct [7]. Secretin MRCP has been used as a problem-solving tool in pancreatic duct anatomy and integrity, and also to assess whether there is ongoing leakage from the pancreatic duct [7,8]. ERCP can diagnose duct disruption and ongoing leaking, and can also be therapeutic in certain clinical scenarios, such as placement of a pancreatic duct stent [9]. However, ERCP is invasive and there is a risk of complications, including pancreatitis [9]. Despite the availability of these diagnostic tools, direct visualization via laparotomy may be required for a definitive diagnosis.

Although serum amylase and lipase are not always elevated in the setting of pancreatic trauma, up to 82% of patients with pancreatic injury will demonstrate an elevated amylase level [10]. In one study, where serial evaluation of serum amylase was performed, sensitivity increased to 90% [10]. Sensitivity of serum lipase in the adult population is not well known, but one study showed that 80% of patients with abdominal trauma demonstrated elevated lipase levels on initial investigation, with increasing levels seen on serial evaluation [10]. Our patient had an elevated serum amylase on blood work taken during her course in our intensive care unit, but her serum lipase was normal. However, serum amylase is not specific to pancreatitis, and can be seen in numerous other conditions, including cerebral trauma, which was present in this patient. As well, our patient’s serum lipase was normal, and given that the negative predictive value of serum lipase has been estimated to range from 94% to 100% [11], a diagnosis of traumatic pancreatic injury in our patient is unlikely. Thus, the pancreatic enlargement and peripancreatic fluid may have been due to another mechanism, such as systemic inflammatory response with cytokine production and release, perhaps related to the massive head trauma. These cytokines, in addition to complement system activation and other mediators, cause increased permeability of the vascular endothelium, which then results in tissue edema. These same mediators also alter cell membrane permeability, which can then cause cell swelling and rupture [12].

Pancreatic injuries are readily diagnosed at exploratory laparotomy [5]. Our patient, as an organ donor, underwent a laparotomy for organ harvesting. The transplant surgeon noted that her pancreas appeared edematous, but there was no evidence of traumatic injury to the organ, and no other signs indicative of trauma within the remainder of her abdominal cavity. The mechanism of injury and distribution of injuries sustained by our patient would make traumatic pancreatic injury highly unlikely. The history and physical examination suggested that a metallic object had struck our patient’s head directly, with no contact to any other part of her body, and nor did she experience blunt trauma from a steering wheel or front-end collision.

Another study documented peripancreatic fluid as a CT sign of shock in patients who sustained abdominal or pelvic trauma, in conjunction with clinical evidence of hypovolemic shock, but without evidence of pancreatic injury [13]. Shock pancreas is a well-documented entity, again seen mainly in the setting of hypovolemic shock, often due to traumatic injury [13-15]. It is thought that this is secondary to fluid shift across compartments, from the intravascular structures to the extravascular or extracellular spaces. This may be directly due to blood loss with replacement by hypo-osmolar fluids, or secondary to cytokine production and release, resulting in increased endothelial and cellular membrane permeability [13]. A similar entity, shock bowel, with marked mucosal enhancement and submucosal edema, is also usually seen in the setting of hypovolemic shock. However, it has now been described in patients who were not hypovolemic, or have not sustained significant blood loss. Shock bowel has been in documented in other clinical scenarios, including isolated head injury, sepsis and recent surgery [14]. It is possible that the pancreatic findings in our patient could represent shock pancreas secondary to neurogenic shock, although as far as we know, this particular entity has not been previously described. The significance of the elevated amylase but normal lipase level in our patient is uncertain. Some studies have documented normal pancreatic enzymes in patients with shock pancreas [13], while other patients demonstrate abnormally elevated amylase and lipase levels [16,17].

Finally, other considerations for imaging findings would include acute pancreatitis secondary to other etiologies. Given that ultrasound is the modality of choice for gallstone imaging, no gallstones were seen on CT, nor was there any intra- or extrahepatic biliary duct dilation. As previously stated, given our patient’s serial lipase levels remained within normal limits, in conjunction with the high negative predictive value of serum lipase for acute pancreatitis, this is an unlikely consideration.

## Conclusion

We present a case report of a patient who presented to our emergency department with an isolated blunt head injury, but with an unexpected finding of an abnormal pancreas on CT, which demonstrated marked edema and peripancreatic fluid. There was no evidence of traumatic pancreatic or other intra-abdominal injury on clinical examination, laboratory values or direct inspection during surgery. The etiology of this finding could not be determined, but possible diagnoses include edematous pancreas secondary to aggressive fluid resuscitation or, alternatively, shock pancreas secondary to neurogenic shock. These imaging findings can also be seen with traumatic pancreatic injury and acute pancreatitis, but this is much less likely here, given the mechanism of injury, blood work and findings at laparotomy.

The increasing use of CT in the evaluation of patients who have sustained trauma has led to increasing detection of unusual and unexpected findings, which can lead to diagnostic dilemma. As our experience with this case demonstrates, the integration of clinical history, blood work and careful evaluation of ancillary imaging findings can be useful in distinguishing between the entities of shock pancreas, overhydration, pancreatitis and traumatic pancreatic injury.

## Consent

Written informed consent was obtained from the patient’s next of kin for publication of this case report and accompanying images. A copy of the written consent is available for review by the Editor-in-Chief of this journal.

## Abbreviations

CT: Computed tomography; ERCP: Endoscopic retrograde cholangiopancreatography; MRCP: Magnetic retrograde cholangiopancreatography.

## Competing interests

The authors declare that they have no competing interests.

## Authors’ contributions

AC interpreted the CT study and acquired further clinical information and follow-up results. AC and EL were the major contributors in writing the manuscript. Both authors read and approved the final manuscript.
